# The Wassermann Reaction

**Published:** 1919-02-15

**Authors:** 


### THE WASSERMANN REACTION.

### Simple Aspects of its Application and Reliability.

Probably no diagnostic test has ever received such world-wide attention or inspired so much rapid research as that originating in the discovery of the Bordet-Gengou phenomenon in 1901, for within five years of that time Wassermann, in conjunction with Neisser and Bruck, had shown that the principle was applicable to the diagnosis of syphilis, and so gave his name to a now famous serological reaction.

Immediate improvements and modifications followed, yet the value of the reaction has repeatedly become surrounded by doubt. Time must always elapse before a busy medical world can come by all the available facts, and so judge the case for itself, but at the moment, when so much is heard and written of venereal disease, its treatment and its effect upon the economy of the nation, we must not turn from a method because in exceptional circumstances it has been known to fail, but rather attempt to blend it with our other available means. Therefore it is the aim in this article to sketch briefly, in relation to the accepted mechanism of venereal treatment, the value of this famous reaction, the principles of its application in a venereal disease clinic, and to indicate some at least of the more important phases in which, as a real aid to diagnosis and control, it reaches its maximum utility.

### The Latest Official Publication.

This book consists of reports of the Special Committee upon the Standardisation of Pathological Methods, No. 4, and is devoted to this one test entirely. It appeared desirable to arrange a definite trial in which a series of specimens from patients of known clinical history should be examined by skilled observers of recognised experience, using similar fractions of serum in each case, and employing the original Wassermann test with its full controls. Dr. Sequeira, of the London Hospital, offered his co-operation, and selected some hundred cases, both syphilitic and non-syphilitic, of ideal variety and completeness of history, retaining himself all clinical information regarding them. The sera, divided into three equal parts, and labelled only with numbers, were sent to three established experts, namely, Colonel L. W. Harrison, Dr. Carl Browning, and Dr. James McIntosh, each worker employing the full Wassermann technique. Their results show remarkable consistency, both in relation to each other and to the cases already decided as syphilitic, and it will be well to quote the more important of the committee's conclusions.

"Upon the study of all the observations presented in this report, the committee consider that in the hands of those whose previous record entitles them to be considered as experts, the percentage of positive Wassermann reactions in active syphilis is so high that the test may, for all practical purposes, be looked upon as specific. It must not be forgotten that the performance of this test involves one of the most complicated methods that have been applied to diagnosis in medicine, a fact which has already been emphasised by the committee in their former report on its technique. Practised as it has been by all sorts of laboratory workers, it was only to be anticipated that in some instances at least inaccurate or fallacious results should have been obtained. Such failures have in certain quarters led to a scepticism with regard to the results, or even to the value of the method itself. Much of the criticism and sceptism, however, has been based on mere statement, and a searching inquiry into the literature of the last eight years reveals, in the opinion of the committee, really very little that can be classed as proved inaccuracy. There is, indeed, a much greater volume of published evidence in favour of the Wassermann test."

### Cases met at Venereal Clinic.

Broadly speaking they will fall naturally into two main classes. The first comprises those who have realised the nature of their disease unaided, particularly in the primary stage, and those who have been at once diagnosed by the first doctor consulted, who therefore sends them direct to the clinic. The second contains the more obscure conditions decided by other departments to represent either early syphilis with unusual sites or manifestations, or else late syphilis in any of the various conditions which may be found among the out-patients of the physician or the dermatologist. Well developed secondary symptoms may make uncertainty impossible, but with the typical cases and primary conditions efficiency can be marred either by unnecessary speculative treatment or by delay until a definitely possible W.R. is established. When a patient, having few other signs, presents a sore and a supposed infection period of one to two weeks, it is a fully accepted fact that the W.R. will probably be negative whether the chancre be syphilitic or not; and also, as frequently happens, the person may have been given with every good intention an intra-muscular dose of mercury some days before his arrival at the clinic. This, again, will largely vitiate the test, if not temporarily producing a complete negative. Hence for rapid work we must here give precedence to a more immediate method, namely, the direct recognition of the casual organism in serum from accessible lesions, and this may be done on the spot with a high-power microscope and dark-ground illumination. The spirochaete or *Treponema pallidum* can be observed with considerable certainty. It may not be out of place roughly to indicate one very excellent type of clinic arrangement, consisting of two small communicating apartments opening into the consulting-room. The one is fitted as a dark room with dark-ground microscope adjusted and always ready, the other has water supply, a stock of collecting apparatus to hand, chair and screen accommodation for the

**The Wasserman Reaction**—(*continued*).

patient, and the whole in charge of a skilled assistant who attends throughout the sitting. A person can be immediately ushered into the collecting-room, the lesion cleaned, dried with a little alcohol, and the exuding serum subjected to microscopic examination. Meanwhile a sample of blood for a later Wassermann test may be taken in the usual way, and is thus obtained before any treatment can have impaired the value of the result. The dark-ground field may give the diagnosis at once, saving many hours, while the W. R. obtained with the whole batch on the next day becomes confirmatory.

### Late Syphilis.

Turning next to the later phases of the disease—that is, to the type common among those forwarded from other medical out-patients—we see the W.R. come progressively into greater importance. Instead of being distributed over the whole system the organism is scarce, and can rarely be detected in any accessible lesion, for the spirochaetes tend to localise themselves scantily in one or more foci, corresponding to which are found the isolated gummata, possibly breaking surface and giving rise to common manifestations on skin, tongue, palate, periosteum, etc., or else remaining invisible, but producing symptoms due to enlargement, pressure, or necrosis. One can only touch upon so tremendous a subject as this, but the point must be made that, given even expert medical opinions, there is no doubt that the best deciding test in these instances is the Wassermann reaction.

Taking an average from well-recognised statistics over a number of years, one arrives at a figure of about 89 per cent. positive reaction in all cases accepted as certain tertiary syphilis, while in more recent times (1912), testing 372 cases which, unknown to them, were diagnosed as clinically certain, Fildes and McIntosh obtained at the London Hospital a result of 97 per cent. positive for tertiary lesions.

### Congenital Cases.

In dealing with the congenital type all observers agree in finding just as high a positive percentage, but in this instance the medical history of the parents and the familiar stigmata make a very characteristic clinical picture, and such are not the cases where laboratory investigation is all important. It is for the very young baby, of known or suspected syphilitic heredity, but as yet showing no signs of disease, that one cannot over-estimate the value of the test. Was the parent infected before or after conception or birth? Is the child the subject of latent syphilis or has it escaped? The test, repeated at intervals if necessary, can provide information of the greatest worth and give a timely warning which may save the young body from ruin.

### Parasyphilis.

And, lastly, of parasyphilis, the same statistics show a very high relative percentage indeed, in fact about 80 per cent., but among the remaining 20 per cent. are undoubtedly cases not of syphilitic origin, for these types of chronic action can be very closely simulated by many other causes of slow pathological change. The diagnosis will practically always be made by the physician, while the serologist will be set the task of determining the true cause of the sclerosis, and apart from his own experience of the small points peculiar to the specific variety, the Wassermann reaction must prove his chief claim to decision.

### Use in Treatment Control.

The degree, routine, and extent of the recognised courses of treatment have been, of late, so widely and frequently published that it is unnecessary to give details here, but supposing that the needed work has been done and the patient apparently recovered, it must be recalled that there may be some small corner unscavenged, as too frequently proved by an unexpected relapse. The obvious control is the serological test detecting all but the minutest residue, but a great fallacy may arise from the effect of the curative agents incompletely eliminated from the blood-stream. There is no doubt that both mercury and arsenical injections can produce a spurious negative reading, the former being by far the worst offender, and though knowledge of the state of the particular case may allow the pathologist to modify his technique and standardisation to counteract this tendency, yet it is one of the commonest indirect causes of relapse when specimens are sent to a distant laboratory and cure assumed on the strength of a misleading Wassermann test.

Even later, to allow for the possible residual organisms, blood must be taken again at intervals up to six months, and even for certainty up to one year after all treatment has ceased. One is apt to forget that, had we not this means, during that six months or year we should be more than a little in the dark, and it is at this stage that the reaction, when properly applied, may be said to reach its maximum economic utility.

### Incidence in Population.

To come finally to one other general consideration, it may be taken as a well-known fact that there are many undoubted instances of the disease; yet showing no symptoms of syphilis, but in which the W.R. is positive. The examinations of such cases have been utilised to determine the incidence of syphilis in the general population. It is unfortunate that there is a fallacy in that these "populations" were not general but selected—usually a hospital population—but it is interesting to note that among a large number of patients, visiting London hospitals for reasons wholly unconnected with syphilis, as high a figure as 8 per cent. is given. Regrettably, when we set about collecting specimens generally from both the fit and unfit, the necessary procedure is far from popular with the patients themselves, and so such investigations have had their limitations, but the instance is given as yet another use of the Wassermann reaction in the approaching conflict with venereal disease.

